# Exploring resilience among hospital workers: a Bayesian approach

**DOI:** 10.3389/fpubh.2024.1403721

**Published:** 2024-08-29

**Authors:** Laura Uccella, Ilenia Mascherona, Sebastiano Semini, Sara Uccella

**Affiliations:** ^1^Department of Emergency, EOC, Ospedale Regionale di Lugano, Bellinzona, Switzerland; ^2^Department of Neurosciences, Rehabilitation, Ophthalmology, Genetics, Maternal, and Child Health (DINOGMI), University of Genoa, Genoa, Italy; ^3^Child Neuropsychiatry Unit, IRCCS Giannina Gaslini, Genoa, Italy

**Keywords:** resilience, healthcare professionals, healthcare providers, personality traits, hospital, physicians, nurses

## Abstract

**Background and importance:**

Healthcare professionals face significant workloads, as their roles are among the most demanding and stressful. Resilience serves as a crucial factor in helping them cope with the challenges encountered in their work environment and effectively manage stress. Assessing the level of resilience among healthcare workers and identifying potential variations across different groups is essential for effective public health management, preventing burnout, and ultimately enhancing patient care.

**Objective:**

To assess the resilience of various categories of workers operating within a tertiary care multisite hospital and understanding if there are any differences in resilience, based on their characteristics, the type of department they work in, and personality traits.

**Design, setting and participants:**

This was a cross-sectional study conducted in January 2024 at EOC, a multi-site tertiary care hospital located in Southern Switzerland. 1,197 hospital workers answered an online survey which included: (1) an *ad hoc* questionnaire on personal and job characteristics, well-being-related activities, satisfaction level regarding communication, collaboration, support, and training opportunities in the workplace, (2) the Connor-Davidson Resilience Scale 10-Item on resilience, and (3) the Big Five Personality Inventory 10-item on personality traits.

**Outcome measures and analysis:**

Proportion of resilient and highly resilient individuals within the various categories of workers were analyzed with Bayesian approach and Bayesian robust regression.

**Main results:**

Being part of the hospitality staff, working as a doctor, and having a male sex were associated to the highest scores of resilience. Surgery and emergency departments had the highest proportion of highly resilient individuals. Male sex, older age, seniority, higher hierarchical rank, engagement in physical activities, relaxation or mindfulness practices, religiosity, perception of good collaboration, communication, support, and physical activity correlated with higher resilience skills.

**Conclusion:**

This cross-sectional study found that physicians and hospitality staff within our multi-site Swiss hospital are more resilient compared to other categories of hospital workers, and among departments, those working in surgery and Emergency Medicine. Enhancing our comprehension of resilience is crucial for more precise management of healthcare systems and the development of employment policies aimed at sustaining the capacity of healthcare systems to serve patients effectively, while also mitigating shortages of healthcare professionals.

## Introduction

Resilience reflects the ability to bounce back after adversity (1–3). It is a dynamic process that can evolve over the course of life and vary depending on the circumstances (1, 4).

It correlates with mental health and well-being (5) as well as with work performance (6) and is influenced by both individual factors (such as personality traits and socioeconomic status) (7, 8) and other variable factors. These variable factors, which can be cultivated, encompass demographic characteristics (such as age, marital status, and level of education), practices of relaxation or mindfulness, engagement in physical activity, as well as aspects of well-being such as sleep quality, work-life balance, and the availability of social support (4, 9–17).

In the workplace, effective collaboration and communication within the team, coupled with organizational factors (favorable shifts, adequate staffing, etc.) and opportunities for teaching technical and non-technical skills, are known to foster resilience. This is particularly true for healthcare workers (4, 18–23).

Healthcare workers are a highly at-risk category to adversities and stressful/traumatic condition, both acutely and chronically, due to their constant exposure to people sufferance, necessity of rapidly responding to requests for help, and the possibility of severe adverse disease outcomes (24).

In recent years, there has been a noticeable shortage of healthcare professionals across all high-income nations, primarily due to many doctors and nurses leaving the profession. The trend is primarily driven by high rates of burnout and challenges in balancing work and personal life. While the COVID-19 pandemic and the climate change related events may have exacerbated this situation, early signs of these issues were present prior to their occurrence (24–33).

This has prompted the proposal and development of various interventions aimed at supporting healthcare staff in self-care and also adjustments in medical education programs (25, 29, 34, 35).

For these reasons, resilience emerges as a highly desirable characteristic in healthcare staff, as it may serve as a protective factor against abandoning the profession.

There is limited and fragmented literature on the resilience levels of healthcare personnel, often encompassing only some aspects of this complex construct (4, 19, 20, 22, 23, 33, 36, 37).

Indeed in some healthcare sector a higher level of intrinsic resilience seems to be necessary. For instance, critical care or surgery departments have the highest rates of workers burnout (24, 37–40).

In line with the demand-resources job model (28), these departments entail high demands (night and weekend shifts, highly stressful and emotional situations) while resources remain similar to those available to other healthcare professionals (12).

There is, however, only little information on this topic (37, 41) and there is not a clear understanding of the level of resilience required for these roles. Understanding of clinician resilience has predominantly stemmed from convenience samples of organizations and clinicians, frequently through surveys targeting either physicians or nurses exclusively.

A hospital is a unique environment where various categories of workers, not just healthcare providers, coexist, allowing for potential differences to be assessed based on job type. Within the same setting, there are roles that inherently require the ability to handle extreme situations, while others do not. Despite numerous confounding factors, several variables such as differences in location, administration, and working conditions are eliminated.

Aim of this study was to assess whether there are differences in resilience among various categories of workers within a hospital, and among healthcare providers.

## Materials and methods

### Ethics statement

This cross-sectional study was conducted in accordance with the Declaration of Helsinki. The protocol underwent review and was deemed exempt by the Ethical Committee of Canton Ticino and the participating hospitals. Participants were invited via email to take part and provided with information regarding the study’s objectives, design, voluntary participation, and the confidentiality of responses. Completion of the survey implied explicit informed consent as participants had to express their consent before starting the questionnaire and it was impossible to fill it in without giving explicit consent. The questionnaire was anonymous: inserting personal data that could lead to individual identification was not required and the responses were only visible to investigators. The study adhered to the Strengthening the Reporting of Observational Studies in Epidemiology (STROBE) reporting guidelines for cross-sectional studies.

### Study design, data collection, and sample

The participants were employees of EOC (Ente Ospedaliero Cantonale), a multi-site hospital located in Canton Ticino, southern Switzerland. EOC comprises three hospitals spread across three different cities, all operating under a unified administration. These hospitals encompass all surgical and medical specialties, as well as critical care area, that is Emergency Department (ED), anaesthesia, intensive care units.

Data collection took place between the 23rd of January and the 6th of February, 2024.

The study entailed the distribution via email of electronic surveys completed by 1,197 respondents (out of 3,907 individuals invited via email), resulting in a response rate of 30% (specific rate for physicians 43% - 277/634, nurses 35% - 455/1283, administrative personnel 38% - 203/567, technical personnel 20% - 137/654, hospitality staff 21% - 54/249, medical practice assistants 15% - 50/322, other 10% - 21/198). Attendance was voluntary and anonymous.

Each respondent completed a three-part questionnaire: the first part (see Supplementary material) was an informative part in which subsequent data was collected:

- Biographical data (age, gender, marital status).
- Job-related data (profession, department, seniority, hierarchical role).
- Well-being related data (physical activity; religiosity; mindfulness or other meditation/relaxation practices).
- Job satisfaction data (satisfaction with time-schedule; perceived collaboration, communication and support by colleagues, work-life balance).
- Data on continuing education (organization of sessions, debriefings).

The second and third part consisted of two validated questionnaires: the Italian version of CD-RISC 10 (Connor-Davidson Resilience Scale) (42) and the Italian version of BFI-10 (Big Five Inventory) (43), which assessed resilience and personality traits, respectively.

### Measure of resilience

The CD-RISC 10 (42) is a tool used to measure resilience, primarily focusing on hardiness. It comprises 10 statements that reflect various aspects of resilience:

Flexibility (items 1 and 5).

Sense of self-efficacy (items 2, 4, and 9).

Ability to regulate emotion (item 10).

Optimism (items 3, 6, and 8).

Cognitive focus/maintaining attention under stress (item 7).

Each statement is rated on a five-point scale from 0 to 4, where 0 indicates the statement is not true at all and 4 indicates it is true nearly all the time. The total score is obtained by summing up all 10 items, resulting in a score ranging from 0 to 40. Higher scores indicate greater resilience, while lower scores suggest less resilience or more difficulty in bouncing back from adversity.

The Connor-Davidson Resilience Scale is one of the most widely used resilience scales in literature. Initially developed for patients with mental disorders, particularly PTSD and anxiety, it has subsequently been demonstrated to have convergent and discriminant validity and reliability across multiple nationalities and populations (12, 44).

The Italian version of the CD-RISC 10 has good psychometric properties, namely reliability and validity, as detailed in the original article (45).

The scale may be insensitive in detecting very high levels of resilience due to a “ceiling effect” toward the upper end. On the other hand, if discrete levels of resilience are detected toward the upper end, the data is reliable (46).

### Analysis of personality traits

The BFI-10 is widely represented in studies focusing on personality and resilience. There are many studies that use it to assess which personality traits are associated with greater resilience (46, 47). Specifically, it appears that neuroticism is negatively correlated with this characteristic, while the other four personality traits (extraversion, agreeableness, conscientiousness, openness to experience) are linked to higher levels of resilience.

### Statistical analysis

Statistical analysis was conducted using several open-source Python packages, including “Bambi,” “Pandasql,” “NumPy,” “PyMC,” “Seaborn,” “Notebook” and “Matplotlib,” with versions 0.13.0, 0.7.3, 1.25.2, 5.10.3, 0.13.2, 7.0.7 and 3.8.2, respectively, on Mac OS 13.4.1. Statistical significance was determined based on highly credible intervals of parameter estimates, with confidence intervals (CI) calculated at 95%.

A Bayesian approach was employed, with uninformative priors, which does not suffer from the sample size limitations inherent in frequentist methods relying on asymptotics. The Bayesian approach addresses uncertainty by generating wider confidence intervals: narrower when data are abundant and wider when data are scarce. Statistical significance is indicated if the confidence intervals of two different subgroups (e.g., young vs. old) do not overlap, as observed in the second communication study (48).

To assess potential differences in resilience among the various groups under examination, we considered participants with high resilience scores (36–40, fourth quartile) and those with low resilience scores (0–25, first quartile) in the various profession and departments.

We then analyzed this highly resilient subjects with respect to all the variables examined.

To assess the solidity of our findings across different model specifications we conducted a Bayesian robust regression analysis, with the goal of assessing credible intervals for each parameter and evaluating the direction of their overall contribution to the CD-RISC 10 score.

We also performed a similar Bayesian regression analysis to determine potential influence of various personality traits on resilience.

## Results

The total sample comprised 1,197 participants, 842 (70.3%) female and 355 (29.7%) male subjects, all collaborators of EOC. The response rate was 28% (1,197/4275 emails sent).

Table 1 describes the responses given with mean CD-RISC score.

**Table 1:** Sample description.

Participants (1197)	No.	%	CD-RISC mean score
**Gender**			
Male Female	355 842	29.7 70.3	31.6 30.4
**Marital status**			
Single Not single	376 821	31.4 68.6	30.8 30.6
**Age (years)**			
18–25 26–30 31–35 36–40 41–45 46–50 51–55 56–60 >60	50 151 205 171 167 143 150 104 50	4.2 12.6 17.1 14.3 14 11.9 12.5 8.7 4.7	30.1 30.0 30.3 29.8 31.0 31.5 31.7 31.2 31.3
**Seniority (years)**			
0–5 6–10 11–15 16–20 >20	278 237 180 152 350	23.2 19.8 180 152 3.9	30.4 30.2 30.5 30.4 31.6
**Role (hierarchy)**			
Executive Manager Worker	168 322 707	14 26.9 59	33.2 30.9 29.9
**Profession**			
Nurse Physician Hospitality staff Administrative staff Technical staff Medical assistant Other	455 277 54 203 137 50 21	38 23.1 4.5 16.9 11.4 4.1 1.7	30.6 31.8 31.7 30.6 30.3 30.0 29.6
**Department**			
Administration Services Outpatient Surgery Critical Area Internal Medicine Other	203 168 108 172 253 255 38	21.1 14 9 14.3 21.1 21.3 3.1	31.4 30.0 30.5 31.8 31.2 30.5 28.6
**Physical activity (times per week)**			
0–1 2–3 4–5 >5	669 429 76 23	55.8 35.8 6.3 1.9	30.2 31.2 32.1 32.7
**Quality of sleep**			
1 - Very poor 2 3 4 5 - Very good	152 264 397 283 101	12.7 22.0 33.1 23.6 8.4	30.0 29.5 30.4 31.9 33.2
**Religiosity**			
1 - Not religious at all 2 3 4 5 - Very religious	480 284 236 119 78	40.1 23.7 19.7 9.9 6.5	30.6 30.7 30.5 30.9 32.2
**Meditation/relaxation practice**			
Never Seldom Sometimes Often Very often	152 264 397 283 101	12.6 22 33.1 23.6 8.4	30.6 30.2 30.9 32.3 32.0
**Mindfulness practice**			
Never Seldom Sometimes Often Very often	855 151 117 58 16	71.4 12.6 9.7 4.8 1.3	30.4 31.0 31.6 32.7 32.9
**Collaboration with colleagues**			
1 - Very poor 2 3 4 5 - Very good	24 82 307 576 224	2 6.8 25.6 48.1 18.7	29.8 28.8 29.5 31.1 32.2
**Communication with colleagues**			
1 - Very poor 2 3 4 5 - Very good	24 109 371 524 169	2 9.1 30.9 43.7 14.1	30.5 28.9 30.0 31.1 32.7
**Support/respect from colleagues (same Dept)**			
1 - Very poor 2 3 4 5 - Very good	24 83 273 529 288	2 6.9 22.8 44.2 24.0	30.2 28.4 29.6 30.9 32.2
**Support/respect from colleagues (other Dept)**			
1 - Very poor 2 3 4 5 - Very good	37 138 418 479 125	3.1 11.5 34.9 40.0 10.4	28.4 28.6 29.6 31.8 33.7
**Work-life balance**			
1 - Very poor 2 3 4 5 - Very good	90 251 401 337 118	7.5 20.9 33.5 28.1 9.8	31.9 30.5 30.7 31.6 33.5
**Technical training**			
1 - Very poor 2 3 4 5 - Very good	147 296 366 280 108	12.2 24.7 30.5 23.4 9	30.5 29 30.7 31.6 33.5
**Non technical training**			
1 - Very poor 2 3 4 5 - Very good	204 313 351 259 70	17 26.1 29.3 21.6 5.8	29.8 29.9 31.0 31.5 32.9
**Organisation of debriefings**			
1 - Very poor 2 3 4 5 - Very good	220 287 311 257 122	18.3 23.9 25.9 21.4 10.1	30.3 29.6 30.9 31.2 32.6
**Organisation of listening moments**			
1 - Very poor 2 3 4 5 - Very good	239 296 324 238 100	19.9 24.7 27 19.8 8.3	30.2 29.5 31.2 31.3 33

Sample description with Connor-Davidson Reilience Scale mean score for every category.

The majority of participants were female (70.3%) and were married or in a stable relationship (68%). All ages from 18 to 65 were well represented, as were seniority and hierarchical roles. 38% of the respondents were nurses.

Male subjects, the older ones, with greater seniority and higher hierarchical rank, turned out to be more resilient. Executives seemed to demonstrate higher levels of resilience compared to both managers and workers, regardless of gender.

**Figure 1:**
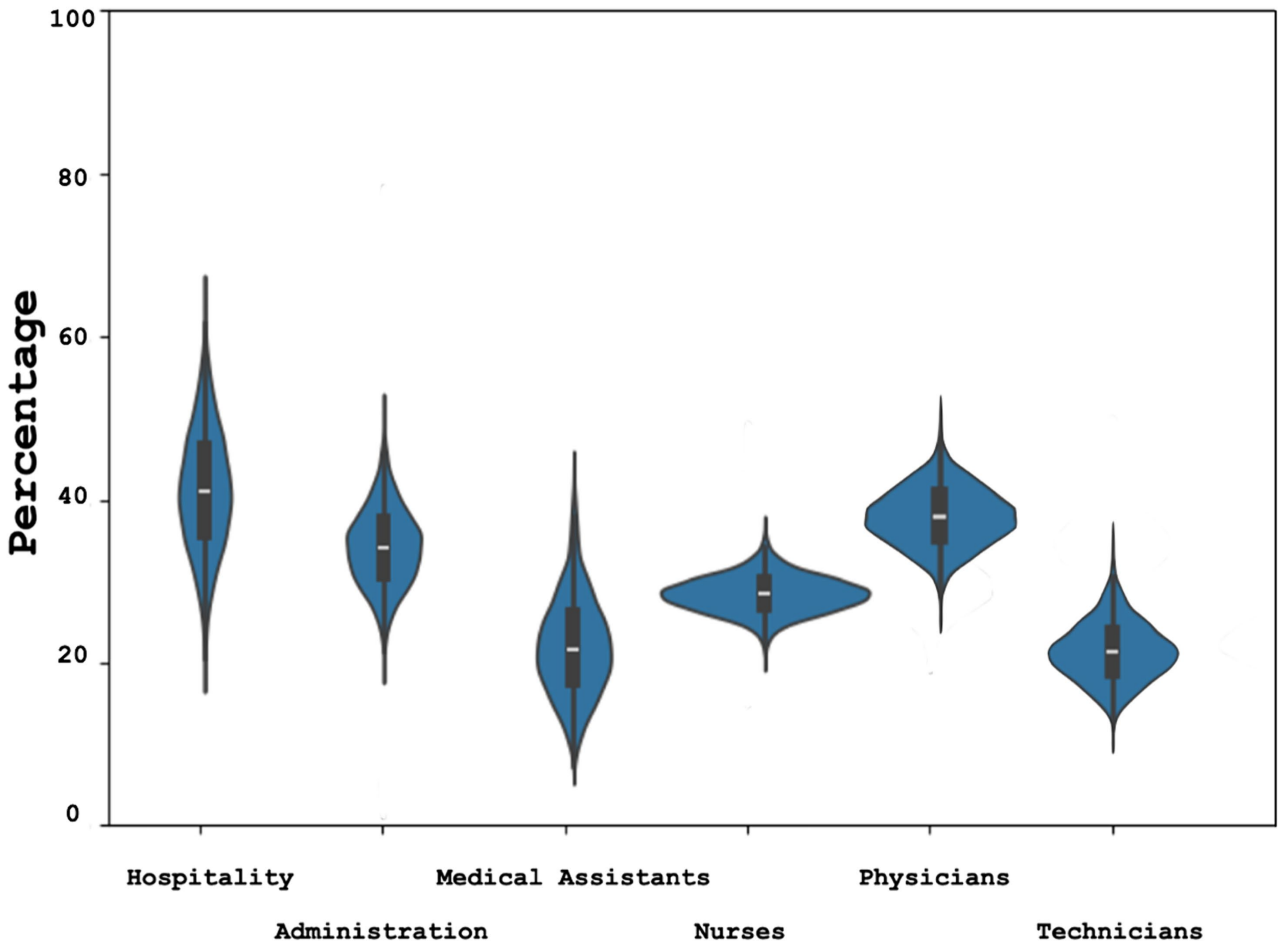
Resilience by profession Bayesian estimate (Confidence Interval) of highly resilient hospital workers estimated with CD-RISC 10 (Connor Davidson Resilience Scale 10 item) divided by profession.

Regarding the profession practiced, respondents who belonged to the hospitality staff and physicians were found to be more resilient compared to other occupations (Figure 1). Moreover, it was observed that surgical departments and the hospitality sector were more resilient.

**Figure 2:**
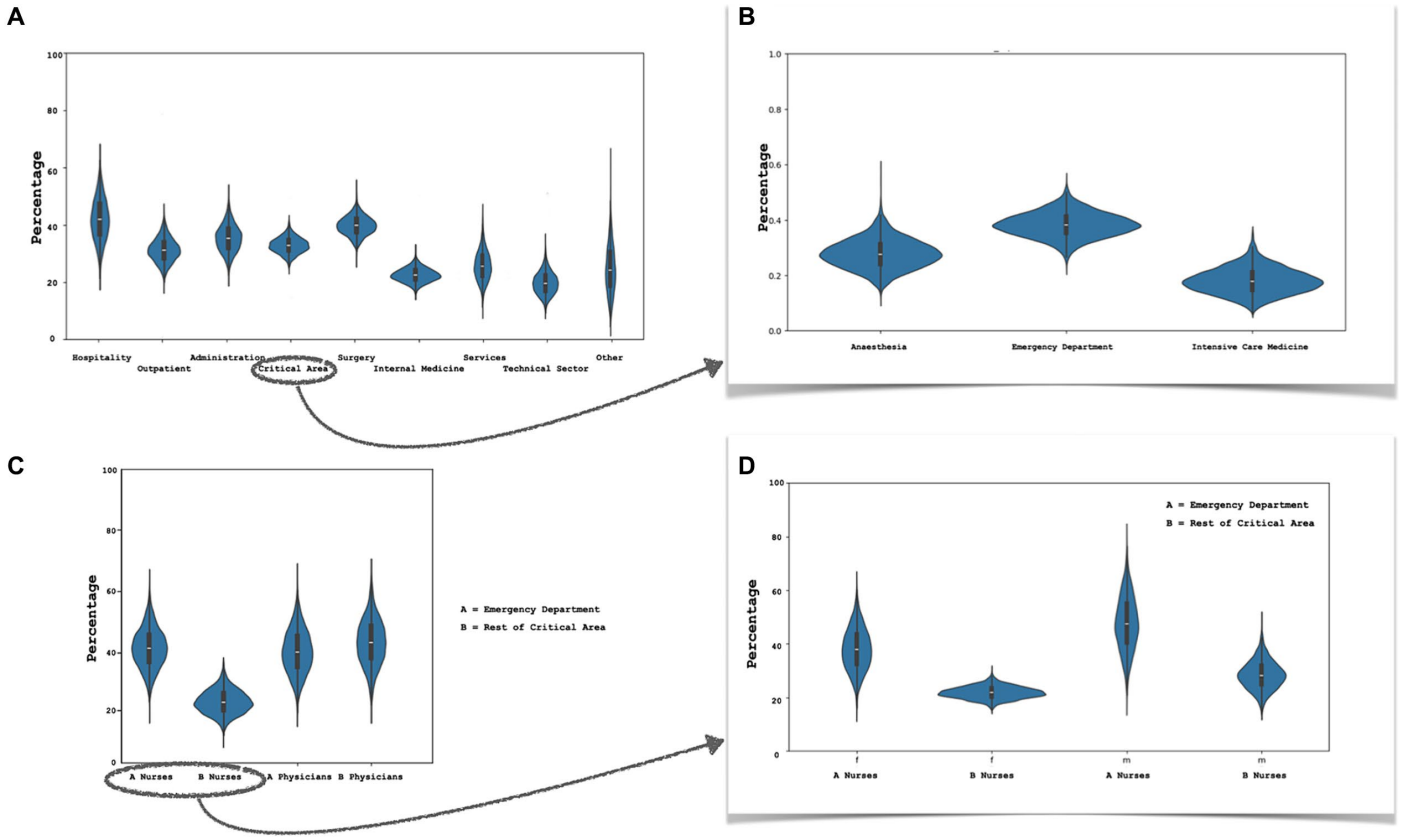
Resilience by department. **(A)** Bayesian estimate (confidence interval) of highly resilient hospital workers estimated with CD-RISC 10 (Connor Davidson Resilience Scale 10 item): **(A)** proportion of highly resilient individuals among the various departments; **(B)** workfers of critical area department; **(C)** comparison between physician and nurses of critical area department: emergency department (A) and the rest of critical area (anaesthesia + intensive care medicine) (B); **(D)** nurses of emergency department compared to critical area (anaesthesia and intensive care medicine) colleagues, sample divided by gender (f, females; m, males).

In the critical care area (emergency department, anaesthesia, intensive care), the department as a whole did not exhibit higher resilience scores. However, when the emergency department was separated, it showed more elevated levels of highly resilient subjects, comparable to those of the surgery departments and the hospitality sector (Figures 2A,B). By further segregating the various professions, it was observed that emergency department nurses displayed a higher proportion of highly resilient individuals, even after accounting for gender differences (Figures 2C,D). The difference was no longer present when considering physicians.

Analysing the factors contributing to well-being, physical activity and mindfulness were found to be associated with higher resilience scores.

The same was true for subjects who self-identified as highly religious.

Regarding collaboration, communication, and perceived support from colleagues (both within one’s own department and from other departments), as well as the opportunity for training both technical and non technical skills, these elements were also associated with higher scores on the CD-RISC.

All these differences were significant after Bayesian regression (Table 2).

**Table 2:** Bayesian robust regression analysis.

		Deviation from Intercept Mean	SD*	HDI 3%	HDI 97%	MCSE^§^ Mean	MCSE SD
Personal data	Gender male	0.936	0.368	0.225	1.600	0.003	0.003
	Marital status single	0.334	0.334	0.783	0.472	0.003	0.003
Profession	Administrative staff	−2.046	1.608	−5.135	0.012	0.029	0.031
	Medical assistant	−1.965	1.782	−5.347	0.424	0.031	0.032
	Physician	−1.001	1.664	−4.181	0.499	0.030	0.030
	Nurse	−1.714	1.635	−4.806	0.032	0.030	0.030
	Technical sector	−3.129	1.664	−6.418	−1.402	0.030	0.030
Hierarchy	Manager	−1.380	0.545	−2.000	−0.329	0.005	0.005
	Worker	−1.834	0.468	−2.250	−1.008	0.004	0.004
Department	Critical Area	0.911	1.700	−2.204	4.176	0.032	0.032
	Surgery	1.794	1.702	−1.274	5.108	0.032	0.032
	Internal Medicine	0.709	1.690	−2.418	3.883	0.032	0.032
	Services	1.176	1.859	−2.227	4.667	0.032	0.032
	Technical sector	0.560	1.790	−2.869	3.857	0.031	0.031
Well being	Physical activity low	0.733	0.335	0.122	1.376	0.003	0.003
	Physical activity medium	1.803	0.651	0.588	2.995	0.007	0.005
	Physical activity high	1.290	1.148	0.858	3.442	0.011	0.009
	Very poor quality of sleep	−0.793	0.549	−1.793	0.289	0.006	0.005
	Poor quality of sleep	0.058	0.516	−0.921	1.024	0.006	0.005
	Medium quality of sleep	1.363	0.562	0.274	2.385	0.007	0.005
	High quality of sleep	2.905	0.718	1.590	4.266	0.008	0.006
	Very poor religiosity	0.053	0.408	−0.7	0.809	0.004	0.004
	Low religiosity	−0.212	0.422	−1.043	0.529	0.004	0.004
	Medium religiosity	0.174	0.558	−0.898	1.215	0.005	0.005
	High religiosity	1.096	0.652	−0.052	2.390	0.007	0.005
	Rare relaxation/meditation	0.326	0.413	−1.100	0.442	0.004	0.003
	Some relaxation/meditation	−0.098	0.499	−0.998	0.863	0.006	0.004
	Frequent relaxation/meditation	0.239	0.746	−1.158	1.650	0.009	0.007
	Very frequent relaxation/meditation	−1.493	1.361	−4.085	0.973	0.015	0.011
	Rare mindfulness	0.916	0.501	−0.033	1.829	0.005	0.004
	Some mindfulness	0.983	0.617	−0.166	2.163	0.007	0.005
	Frequent mindfulness	1.775	0.903	0.065	3.413	0.010	0.007
	Very frequent mindfulness	3.227	1.708	0.129	6.526	0.018	0.013

SD, standard deviation.

HDI, highest density interval.

MCSE, Monte Carlo standard error.

Missing values are the intercept.

The examination of specific personality traits using the BFI-10 indicated that agreeableness, openness to experience, and conscientiousness were positively linked with higher resilience, whereas for neuroticism and extraversion there was a negative association, more pronounced for neuroticism.

**Figure 3:**
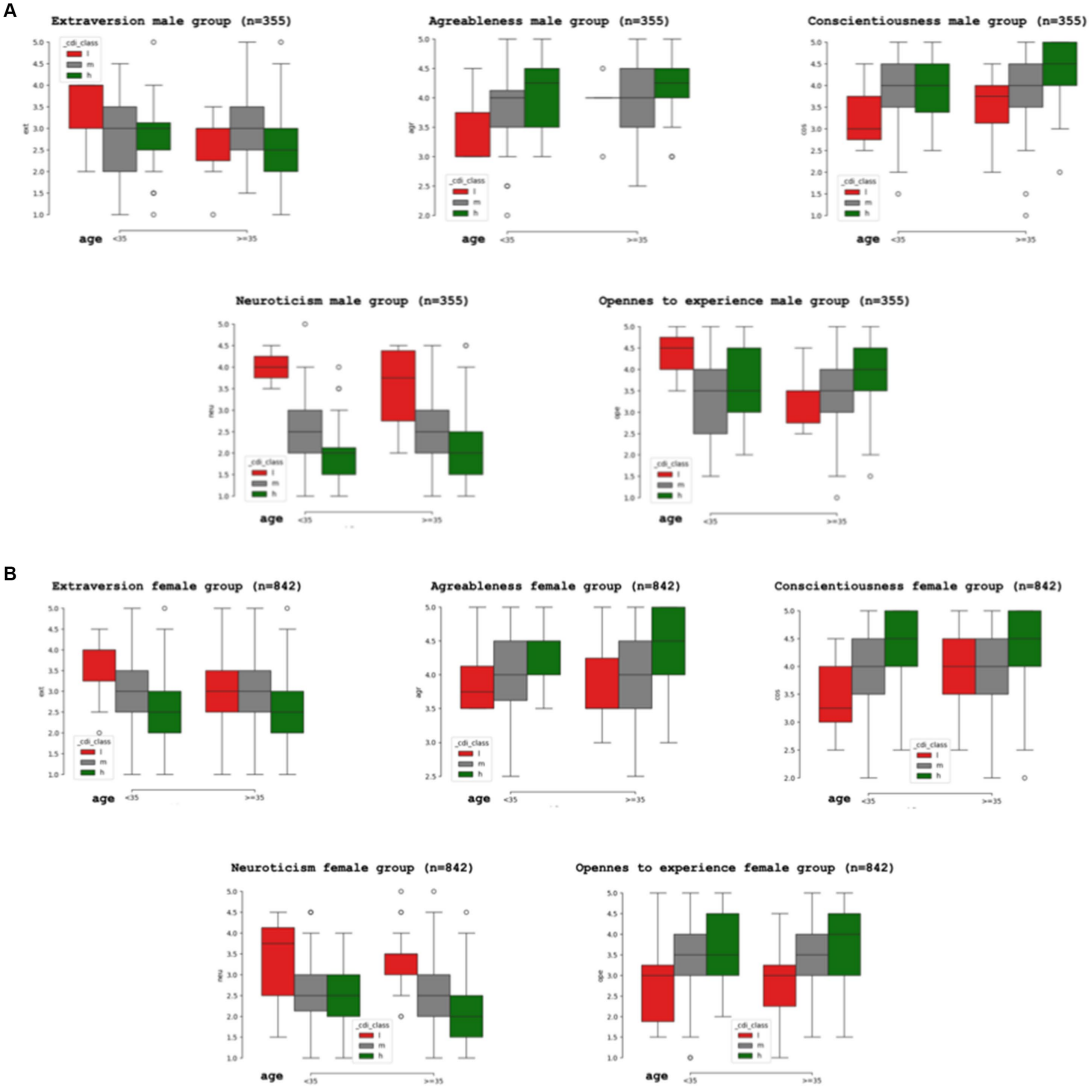
Resilience by gender. **(A)** Proportion (confidence interval) of high (h), medium (m) and low (l) resilience male individuals linked to personality traits assessed with the BFI-10 (Big Five Inventory 10 item): agreeableness, conscientiousness and openness to experience are associated with higher resilience, neuroticism and extraversion are negatively linked to resilience. **(B)** Proportion (confidence interval) of high (h), medium (m) and low (l) resilience female individuals linked to personality traits assessed with the BFI-10 (Big Five Inventory 10 item): agreeableness, conscientiousness and openness to experience are associated with higher resilience, neuroticism and extraversion are negatively linked to resilience.

Since the reference values of the BFI-10 differ for subjects ≤35 years and those over 35 years old, calculations were conducted separately for the two age groups. The participants were segregated by gender due to the higher resilience reported in the male group. Figures 3A,B illustrate the distribution of scores for different personality traits in the male (Figure 3A) and female (Figure 3B) populations, correlated with high (CD-RISC 10 score 35–40), medium (CD-RISC 10 score 25–34), and low (CD-RISC 10 score 0–25) levels of resilience.

## Discussion

In this study, we offer comprehensive evidence of difference in terms of score of the CD-RISC 10 scale and proportion of highly resilient individuals among hospital collaborators based on a large sample of respondents.

Being part of the hospitality staff or working as a doctor, was associated to the highest levels of resilience. Surgery and emergency departments had the highest proportion of highly resilient individuals. Male sex, older age, seniority, higher hierarchical rank, engagement in physical activities, relaxation or mindfulness practices, religiosity, perception of good collaboration, communication, social support, and physical activity correlated with higher resilience skills. The examination of specific personality traits using the Big Five Inventory 10 item (BFI-10) indicated that agreeableness, openness to experience, and conscientiousness were positively linked with higher resilience, whereas for neuroticism and extraversion there was a negative association, more pronounced for neuroticism.

To the best of our knowledge, this is the first study assessing resilience skills on a large sample of hospital workers which includes all job categories and finding that physicians, hospitality staff, and people working in the Emergency Department and Surgery Department are the most resilient hospital workers.

Men appear to be more resilient than women and this in agreement with the literature (49, 50). It’s not clear why this occurs, but it’s known that the stress response differs between males and females, and that women have a higher rate of burnout and anxiety (26, 51). The mechanism with which this happen is obscure, as resilience neural circuits have been only partially identified, with the majority of studies performed on male subjects (52). In a study conducted on rodents, the Authors show how neural reactions are different between male and female subjects (51).

Age, seniority and hierarchical role are predictors of superior resilience, as expected (4, 6, 9, 22). Aging is synonymous with adaptation and experience, not only with senescence (49). At the same time, those who have stayed in a specific position for an extended period and those in leadership roles have likely found the resources to do so, and consequently, they are likely self-selected as more resilient.

Interestingly in our sample there was no statistical difference between male and female executives (females being far less numerous than males).

Hospitality staff and physician were found to be more resilient in comparison to other professionals. There are few studies in the literature on healthcare staff, and even fewer deal with hospitality sector. In particular, the hospitality sector has only recently been studied in relation to the forced closures implemented by numerous governments during the COVID-19 pandemic (52). To become a physician and work in a hospital requires many years of study, while working in the hospitality sector of a health facility means doing humble work, often under-appreciated even by the hospital’s own staff. In both cases, a high level of motivation is required, and perhaps this is why these two categories stand out in the resilience ranking.

It’s possible that motivation also plays a significant role in the presence of highly resilient individuals within emergency departments: working 24/7, overcrowding, and highly stressful situations are part of everyday life, and individuals who are unmotivated or less resilient are not easily found in such environments (53). Unfortunately, we did not assess the motivational aspects and work engagement of our respondents.

Well-being, physical activity and mindfulness as well as the perceived presence of good communication, collaboration and social support among co-workers were all factors that were associated to higher levels of resilience: given that resilience can be nurtured (18, 20–23) clinics’ and hospitals’ administrations should be very attentive to these aspects and develop programs that include a caring vision not only for patients but also for the staff (34, 35, 54). This necessitates a cultural shift that is just starting to emerge within the healthcare sector.

In addition, fostering resilience should involve organising an adequate array of technical and non-technical skills training activities, both of which have been linked in this study to higher resilience scores. Even in this field, studies in the literature are scarce. In this 2022 study (55) involving 111 nursing students, the authors were able to identify a correlation between non-technical skills training and increased resilience, but further studies are needed to confirm the validity of these results.

Finally, there is an evident link between personality traits and resilience, already known in the literature (46) and confirmed by this study, notably the negative correlation with neuroticism (or emotional instability). This highlights implications for both the selection of personnel and the provision of psychological support for healthcare workers.

## Limitations

- Our study has some limitations:
- This paper is based solely on a Swiss context. It is necessary to expand the sample and include other countries in order to generalize the results.
- We did not assess the dynamic aspect of resilience but merely provided a snapshot. It would be advisable to consider evaluating the longitudinal trends in the future.
- The aspect of work engagement was not assessed.

## Conclusion

In conclusion, to our knowledge, this represents the first study that compares various professionals working within the same organization, providing statistically robust data in support of the findings.

The results have implications for enhancing worker resilience, guiding the selection of human resources within a hospital setting, maintaining quality of patients’ care, and stimulating a culture that makes leadership positions accessible to women as well.

## Data Availability

The original contributions presented in the study are included in the article/Supplementary material, further inquiries can be directed to the corresponding author/s.
